# Adult *Opisthorchis viverrini* Flukes in Humans, Takeo, Cambodia

**DOI:** 10.3201/eid1707.102071

**Published:** 2011-07

**Authors:** Woon-Mok Sohn, Eun-Hee Shin, Tai-Soon Yong, Keeseon S. Eom, Hoo-Gn Jeong, Muth Sinuon, Duong Socheat, Jong-Yil Chai

**Affiliations:** Author affiliations: Gyeongsang National University School of Medicine, Jinju, South Korea (W.-M. Sohn);; Seoul National University College of Medicine, Seoul, South Korea (E.-H. Shin, J.-Y. Chai);; Seoul National University Bundang Hospital, Sungnam, South Korea (E.-H. Shin);; Yonsei University College of Medicine, Seoul (T.-S. Yong);; Chungbuk National University College of Medicine, Chongju, South Korea (K. S. Eom);; Korea Association of Health Promotion, Seoul (H.-G. Jeong, J.-Y. Chai);; Centre for Parasitology, Entomology, and Malaria Control, Phnom Penh, Cambodia (M. Sinuon, D. Socheat)

**Keywords:** Opisthorchis viverrini, adult liver flukes, trematodes, parasites, humans, Cambodia, letter

**To the Editor:** *Opisthorchis viverrini* and *Clonorchis sinensis,* the 2 major species of small liver flukes (family Opisthorchiidae), cause chronic inflammation in the bile duct, which leads to cholangitis and cirrhosis of the liver, and are a predisposing factor for cholangiocarcinoma (*1*). Human infections with *O*. *viverrini* flukes are found along riverside areas of Indochina (Thailand, Lao People’s Democratic Republic [PDR], and Vietnam) (*2*).

Small trematode eggs (length 20–32 μm) have been found in human fecal samples in Cambodia (*1**,**3**,**4*). During 1981–1982, two of 102 Cambodian refugees in the United States were found to be positive for *C*. *sinensis* (likely *O*. *viverrini*) eggs (*3*). Egg-positive cases were later detected in several provinces of Cambodia (*4**,**5*). Presence of *O*. *viverrini* flukes in Cambodia was verified by detection of metacercariae in freshwater fish in a lake on the border between Takeo and Kandal Provinces and by isolation of adult flukes in experimentally infected hamsters (*6*).

In May 2010, we analyzed fecal samples from 1,993 persons in 3 villages (Ang Svay Chek, Kaw Poang, and Trartang Ang) in the Prey Kabas District, Takeo Province, Cambodia, ≈45 km south of Phnom Penh, to confirm the presence of *O*. *viverrini* flukes among humans. We found an egg-positive rate of 32.4% for small trematode eggs. Because these eggs may be those of *Haplorchis* spp. flukes (*H*. *taichui*, *H*. *pumilio*, and *H*. *yokogawai*) and lecithodendriid flukes (*Prosthodendrium molenkampi* and *Phaneropsolus bonnei*) (*1*), we attempted to detect adult flukes that are responsible for these eggs.

Six of the small trematode egg–positive villagers, 1 man and 5 women (age range 16–72 years), who had occasional epigastric discomfort were selected for anthelmintic treatment, purgation, and recovery of adult worms. Fecal examination and anthelmintic treatment of villagers were approved by the Ministry of Health, Cambodia, under the agreement with the Korea–Cambodia International Collaboration on Intestinal Parasite Control in Cambodia (2006–2011). After obtaining informed consent, the villagers were treated with a single oral dose of praziquantel, 40 mg/kg (Shinpoong Pharmaceutical Co., Seoul, South Korea), and given a purgament (solution containing 30–40 g MgSO_4_). Feces was obtained 3 or 4 times in a 2–3-hour period after purgation, pooled individually, and processed as described (*7*). Worms obtained were fixed with 10% formalin, stained with acetocarmine, and identified by morphologic features.

A total of 34 *O*. *viverrini* adult worms were obtained from the 6 villagers (14, 9, 5, 3, 2, and 1 from each villager, respectively). No other species of trematodes were obtained. Five worms were lanceolate and had a mean length of 9.5 mm (range 6.5–12.0 mm), a mean width of 1.5 mm (range 1.2–1.7 mm), and 2 characteristic 4–5-lobulated testes (Figure, panel A). Ten eggs in uteri were 27 μm long (range 25–29 μm) and 15 μm wide (range 13–16 μm).

**Figure:**
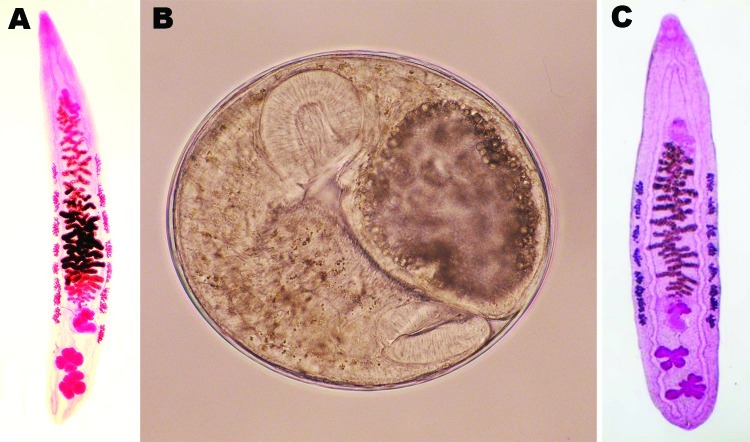
A) Adult *Opisthorchis viverrini* liver fluke (length 12.0 mm) isolated from a human after chemotherapy and purgation in Takeo Province, Cambodia, showing the characteristic morphology of the two 4–5-lobulated testes. B) Metacercaria of *O*. *viverrini* fluke (diameter 0.22 mm) detected in a freshwater fish (*Puntioplites proctozysron*). C) Young adult *O*. *viverrini* fluke (length 5.5 mm) isolated 6 weeks after experimental infection of a hamster with metacercariae from *P*. *proctozysron* fish. Original magnification levels ×8 (A), ×120 (B), ×9 (C).

To detect the source of infection, 2 freshwater fish species, *Puntioplites proctozysron* (n = 5) and *Cyclocheilichthys apagon* (n = 10), were caught in nearby Ang Svay Chek village and examined for *O*. *viverrini* metacercariae by using a digestion technique (*8*). A total of 50 metacercariae (Figure, panel B) were obtained from 5 *P. proctozysron* fish and fed to a hamster. Six weeks later, 42 young *O*. *viverrini* flukes (Figure, panel C) were isolated from the biliary tract of the hamster.

Our study identified only *O*. *viverrini* infections in humans in Cambodia. However, eggs of other hepatic and intestinal flukes also can be found in humans (*1*). In Thailand, Vietnam, and Lao PDR, opisthorchiids (*O*. *viverrini* and *C*. *sinensis*), heterophyids (*Haplorchis* spp., *Centrocestus formosanus*, and *Stellantchasmus falcatus*), and lecithodendriids have been found in humans (*1**,**7**,**9*). In several provinces in Lao PDR, mixed infections with *O*. *viverrini* and heterophyids or lecithodendriids were common (*7**,**9*), and the relative prevalence of each fluke species varied by locality. In Vientiane, Lao PDR, *O*. *viverrini* was the predominant species, whereas in Saravane Province, *H*. *taichui* predominated (*7*). In a mountainous area of Phongsaly Province, *H*. *taichui* and *H. yokogawai* worms were obtained from 10 villagers; however, no *O*. *viverrini* worms were detected (*10*). Thus, in Cambodia, the presence of human infections with intestinal flukes, including *Haplorchis* spp. and lecithodendriids, cannot be ruled out.
